# Response to 1064 nm Nd:YAG LASER in treatment of vulvar Fordyce's Angiokeratoma: case report

**DOI:** 10.1590/1677-5449.202301212

**Published:** 2024-10-07

**Authors:** Cláudia Carvalho Sathler-Melo, Daniel Mendes-Pinto, Guilherme de Castro-Santos

**Affiliations:** 1 Clínica Angiovasc, Nova Lima, MG, Brasil.; 2 Hospital Felício Rocho, Belo Horizonte, MG, Brasil.; 3 Universidade Federal de Minas Gerais – UFMG, Belo Horizonte, MG, Brasil.

**Keywords:** angiokeratoma, Nd-yag 1064 laser, Fordyce, angioceratoma, *laser* de Nd: YAG 1.064 nm, Fordyce

## Abstract

Fordyce's Angiokeratoma (AKF) is a benign type of vascular lesion that affects the male and female external genitalia. Prevalence is unknown and there is no apparent hereditary component. Diagnosis can be suggested by ectoscopy, by the presence of purple, bluish, black or red papules on the vulva, clitoris, scrotum, or penis body or glans. Most patients are asymptomatic. Various methods have been used for treatment, including surgery, electrocoagulation, chemical cauterization, sclerotherapy, and LASER. The effectiveness and complications of these therapies are varied. The purpose of this article is to report the case of a 56-year-old woman with abundant and painful vulvar lesions treated with a 1064 nm Nd:YAG LASER. The 1064 nm Nd:YAG LASER proved to be a viable therapeutic option, with satisfactory results and a low rate of complications.

## Resumo

O angioceratoma de Fordyce é um tipo de lesão vascular de caráter benigno, que compromete as genitálias externas masculina e feminina, cuja prevalência é desconhecida e sem componente hereditário aparente. O diagnóstico pode ser sugerido à ectoscopia pela presença de pápulas púrpuras, azuladas, negras ou vermelhas na vulva, clitóris, escroto, corpo ou glande do pênis. A maioria dos pacientes é assintomática. Vários métodos têm sido utilizados para o tratamento, incluindo cirurgia, eletrocoagulação, cauterização química, escleroterapia e *laser*. A eficácia e as complicações dessas terapias são variadas. O propósito deste artigo é relatar o caso de uma mulher de 56 anos de idade apresentando lesões vulvares abundantes e dolorosas, tratadas com o *laser* de pulso longo Nd: YAG de 1.064 nm. O *laser* Nd: YAG de 1.064 nm apresentou-se como uma opção terapêutica viável, com resultados satisfatórios e baixo índice de complicações.

## INTRODUCTION

Angiokeratomas (AGK) are benign lesions with ectasia of blood vessels in the papillary dermis and hyperkeratosis.^1^ In 1967, Imperial and Helwig^2^ suggested a clinical classification of AGK into five types: angiokeratoma corporis diffusum, with cardiovascular and renal impairment (Fabry’s disease); angiokeratoma of Mibelli, presenting at the extremities of bony prominences; angiokeratoma circumscriptum, with plate shaped lesions, more prevalent in the lower extremities; solitary or multiple angiokeratoma, occurring in any body part; and angiokeratoma of Fordyce (AKF), first described on the scrotum, but also found on both male and female genitals.

In this article, we present the case of a 56-year-old woman with multiple genital lesions on both sides of her vulva and complaining of local pain. She was successfully treated with 1064 nm Nd:YAG Light Amplification by Stimulated Emission of Radiation (LASER), obtaining relief from pain and remission of all lesions in a few months. A literature review of treatment options is also presented.

## CASE REPORT

**Figure 1:**
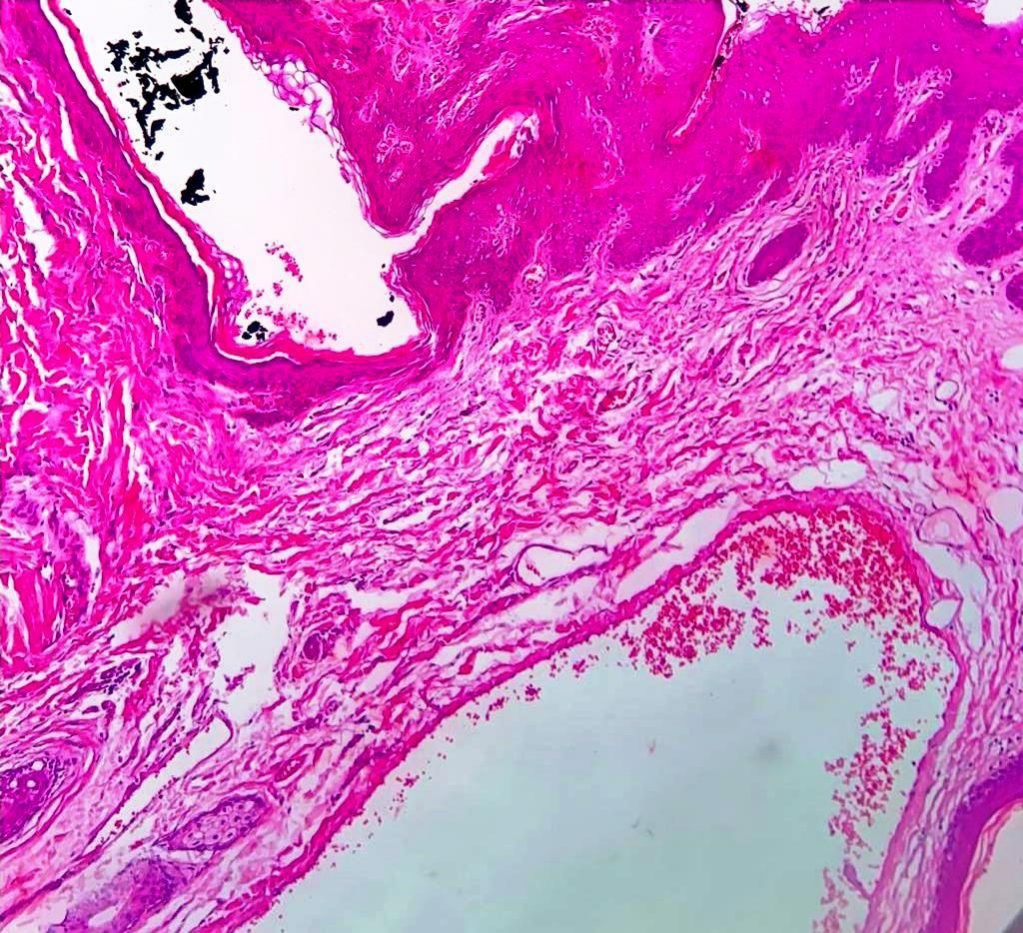
Vascular abnormalities in the papillary derma, ectasia of vessels and hyperkeratosis in the epidermis. Hematoxylin eosin stain (30x).

**Figure 2:**
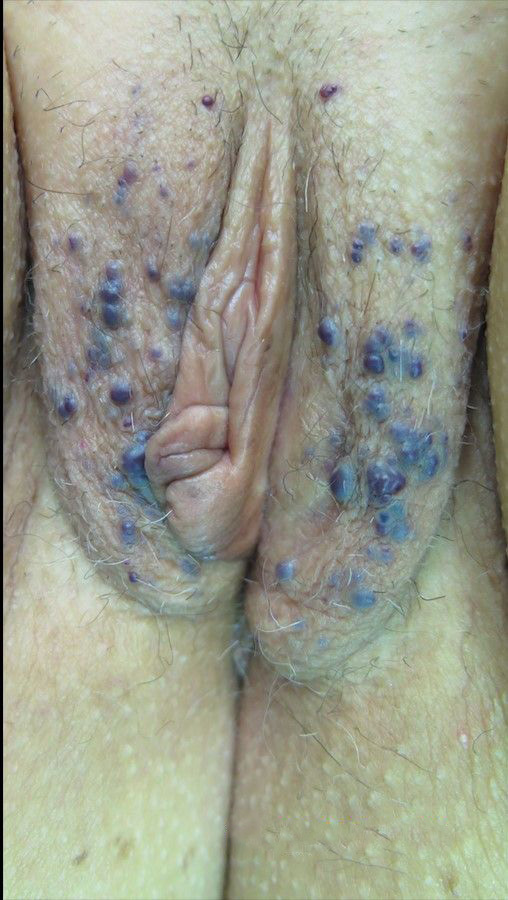
Pretreatment: painful red-purple papules on the vulva.

**Figure 3:**
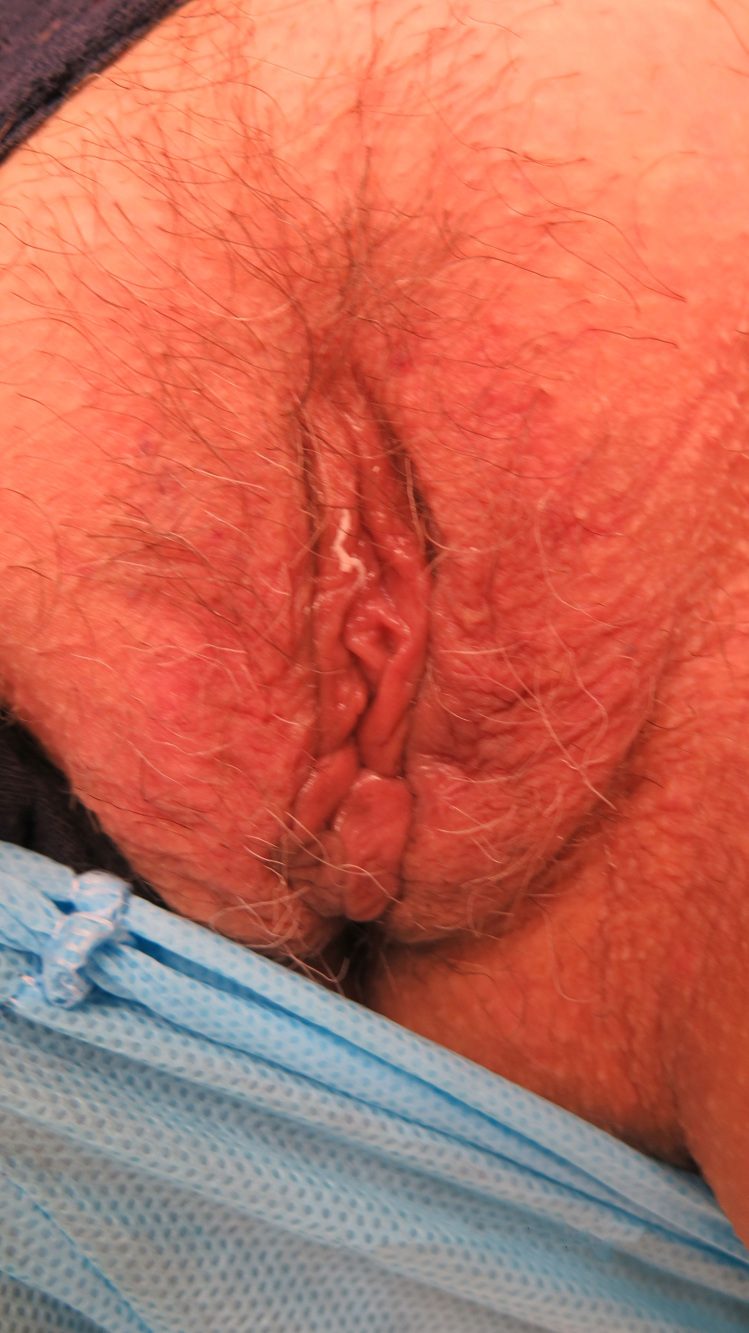
Posttreatment: full remission of lesions and full relief from pain.

This report was approved by the local Ethics Committee, process number: 6130.029. The patient was a 56-year-old white woman with painful red-purple papules on her vulva, bilaterally, for at least 8 years. She went to a gynecologist who treated her with surgical sutures, not described in detail. She had partial remission of the lesions for 3 years, but they relapsed after that time. In 2017, she attended a dermatologist for consultation and a biopsy was taken. The result of the biopsy demonstrated the presence of vascular abnormalities in the papillary derma, meaning ectasia of vessels at this level, as well as hyperkeratosis in the epidermis (Figure 1). There was no malignancy. In the same year, 2017, she was referred to a vascular surgeon because of a suspicion of hemangioma. Pelvic magnetic resonance imaging showed compression of the left common iliac vein between the right common iliac artery and the nearest vertebra. She therefore underwent an endovascular procedure for stenting at the level of compression, with no complications. However, there was no relief from pain nor remission of lesions. In 2020, she presented again with the same complaints, vulvar papules and local pain (Figure 2). Investigation with duplex scanning found no abnormalities at the site of stenting of the left common iliac vein. A diagnosis of AKF was made based on clinical and histopathological findings. The patient was treated with long pulse 1064 nm Nd:YAG LASER on three occasions. In the first session she was given 151 pulses (70 joules/spot size 6 mm/15 milliseconds), under topical anesthesia (lidocaine 7% plus tetracaine 7%). The endpoint was blanching and/or reduction (shrinking) of the lesions. Another two sessions were administered over the next 6 months, with shorter duration and less irradiation, using the same parameters described above. The intervals between sessions were 8-12 weeks. Recommendations for local care after each session were gentle cleaning and reparative ointment (Cicaplast Baume B5 La Roche Posay) twice a day for thirty days. There was full remission from lesions and full relief from the pain, without any significant scars. Results were maintained at 12-month follow-up (Figure 3)

## DISCUSSION

Fordyce’s AKF was first described in 1896 by John Addison Fordyce^3,4^. Typical presentation is the presence of red-purple papules on the scrotum, penile body, glans, clitoris, or vulva. Most patients are asymptomatic. In some cases, there is bleeding, itching or pain. Dermatoscopy is an accurate method for diagnosis of AKF, but histopathological findings are essential to discriminate from other skin lesions, specially malignant ones.

In his first publication, Fordyce^4^ suggested the possibility of high pressure in the pelvic veins as the etiopathogenesis, because his patient, a 60-year-old male also had varicocele. Thereafter, some authors have tried to connect high pressure in the pelvic and abdominal veins with the appearance of the genital papules. In 1989, Cohen et al.^5^ reported the case of a 55-year-old black woman with AKF, hemorrhoids, and uterine myoma. In 2011, Fogagnolo et al.^6^ suggested that the etiopathogenesis of AKF could be related to high local venous pressure and consequently sub epithelial vascular dilation. According to our literature search, to date the etiological origin of AKF is not clear. The higher incidence in older people would suggest a degenerative aspect but this has not been proven yet. In our patient, correction of compression of the left common iliac vein did not yield any clinical improvement or remission of the lesions.

Among the many different treatments, such as surgical excision, electrocoagulation, cryotherapy, sclerotherapy, and several types of LASERs, Ibrahim compared the results of two LASERs: a 595 nm pulsed dye LASER (PDL) and a 1064 nm long pulsed Nd:YAG LASER for treatment of 22 patients with AKF in 2016.^7^ The results of this randomized blinded study revealed statistically significant improvement with both LASERs. However, the 1064 nm long pulsed Nd:YAG LASER was superior to the 595 nm PDL (PDL: 61.8% versus Nd:YAG: 77.63%; p<0.005).

Zeng et al.^8^ admits there is no consensus on the best AKF treatment, but defends the optimal response after treating 11 patients with the 1064 nm long pulsed Nd:YAG LASER. The mean number of sessions was 2.2 and the treatment interval was at least 8 weeks. Ozdemir et al.^9^ mentions collateral effects of other therapies such as lack of uniformity with electrocoagulation and cryotherapy. If there are many papules, surgery is not suitable and application of liquid nitrogen may lead to residual hypopigmentation as well as scaring. Other kinds of LASERs like the 532 nm KTP or ablative LASERs like the Carbon Dioxide LASER and the Erbium-doped YAG have also been used for treating AKF, but efficacy is lower because of the lower capacity of penetration, including high pressure in the abdominal pelvic veins. The concomitant presence of hemorrhoids, varicocele, pelvic varicose veins, or varices in the lower extremities can increase suspicion of that explanation. However, in our case, despite correction of the left common iliac vein compression with an endovascular approach, no improvement was observed. In common with many authors, we had success in clearing all lesions and eliminating complaints of pain in a few months, with no adverse effects.

## CONCLUSION

The 1064 nm long pulsed Nd:YAG LASER was safe and effective for treatment of AKF in this case report.
